# MR habitat radiomics for differentiating indeterminate (Grade 3) nasopharyngeal lesions: lymphoid hyperplasia versus early-stage nasopharyngeal carcinoma

**DOI:** 10.1186/s12885-026-16524-y

**Published:** 2026-07-15

**Authors:** Zhenhuan Huang, Xuezhao Tu, Qikui You, Dandan Lin, Tingyu Yu, Yueming Li

**Affiliations:** 1 Department of Radiology, Longyan First Affiliated Hospital of Fujian Medical University, Longyan, 364000 Fujian China; 2 Department of Orthopedics, Longyan First Affiliated Hospital of Fujian Medical University, Longyan, 364000 Fujian China; 3 Department of Radiology, the First Affiliated Hospital of Fujian Medical University, 20 Cha-Zhong Road, Fuzhou, 350005 Fujian P.R. China; 4 Department of Radiology, Binhai Campus of the First Affiliated Hospital, National Regional Medical Center, Fujian Medical University, Fuzhou, 350212 Fujian China; 5 Key Laboratory of Radiation Biology of Fujian higher education institutions, the First Affiliated Hospital, Fujian Medical University, Fuzhou, 350005 Fujian China

**Keywords:** Nasopharyngeal lymphoid hyperplasia, Nasopharyngeal carcinoma, Magnetic resonance imaging, Habitat radiomics

## Abstract

**Background:**

To investigate the feasibility of MRI-based habitat radiomics to differentiate indeterminate Grade 3 nasopharyngeal lesions into nasopharyngeal lymphoid hyperplasia or early-stage nasopharyngeal carcinoma, and to explore its complementary value for radiologists in diagnostically ambiguous cases.

**Methods:**

In this retrospective study, 138 patients with Grade 3 nasopharyngeal lesions who underwent contrast-enhanced MRI and endoscopic biopsy were included and randomly divided into training (*n* = 97) and test (*n* = 41) cohorts. Radiomics features were extracted from whole lesions and from intralesional subregions generated by clustering analysis. Radiologists’ assessments were performed according to the established MRI grading system. Feature selection was conducted using intraclass correlation coefficient analysis, Pearson correlation filtering, and least absolute shrinkage and selection operator regression. Habitat radiomics, conventional radiomics, and clinicoradiological models were developed using multiple machine learning classifiers. Model performance was evaluated using the area under the receiver operating characteristic curve (AUC), calibration curves, and decision curve analysis. Radiologist performance was assessed with and without model assistance. Model interpretability was explored using SHapley Additive exPlanations (SHAP).

**Results:**

The habitat radiomics model achieved a test-cohort AUC of 0.838, which was numerically higher than those of the conventional radiomics and clinicoradiological models, although the differences were not statistically significant. Radiologist AUCs ranged from 0.632 to 0.828 without model assistance and from 0.828 to 0.928 with habitat radiomics assistance, with significant improvements mainly observed among junior radiologists (*P* < 0.05).

**Conclusion:**

MRI-based habitat radiomics showed feasible performance for differentiating indeterminate Grade 3 nasopharyngeal lesions, although its standalone performance was not statistically superior to comparator models. It may provide complementary assistance for junior radiologists in challenging cases.

**Supplementary Information:**

The online version contains supplementary material available at https://doi.org/10.1186/s12885-026-16524-y.

## Introduction

Nasopharyngeal mucosal thickening on magnetic resonance imaging (MRI) is most commonly caused by nasopharyngeal lymphoid hyperplasia (NPLH) or nasopharyngeal carcinoma (NPC) [1]. Although advanced carcinoma is usually recognizable by deep tissue invasion or mass formation, early-stage NPC typically presents as focal, asymmetric thickening of the nasopharyngeal wall, whereas symmetric thickening favors benign lymphoid hyperplasia [2]. However, a subset of NPLH cases also demonstrates asymmetric mucosal thickening that mimics malignancy, creating a clinically relevant diagnostic challenge.

The MRI grading system proposed by the Chinese University of Hong Kong stratifies nasopharyngeal findings into five categories [3]. Within this system, Grade 3 denotes lesions with indeterminate imaging features, characterized by asymmetric wall thickening or signal alteration without expansile behavior. Importantly, Grade 3 reflects an imaging-based state of diagnostic uncertainty rather than a specific pathological diagnosis. Lesions in this category may ultimately correspond to either lymphoid hyperplasia or early-stage carcinoma at histopathology.

Endoscopic biopsy is the reference standard for diagnosing carcinoma [4]. However, its invasiveness and potential complications limit its application, especially in patients with subtle mucosal abnormalities [5]. MRI plays a central role in initial evaluation. Typical MRI findings of NPLH include vertical striations and symmetric thickening of the nasopharyngeal mucosa and adenoids, nasopharyngeal bubbles, retention cysts, and serrated protrusions [1, 5–7]. These features reflect benign processes such as mucous gland dilatation and adenoidal involution and have been reported to aid differentiation from carcinoma. Nevertheless, asymmetric mucosal thickening has been described in a substantial proportion of NPLH cases, limiting the reliability of conventional imaging markers in indeterminate presentations [1, 4, 5]. Interpretation of Grade 3 lesions depends heavily on the reader’s experience and remains subject to interobserver variability. Recent advances in radiomics and deep learning allow the extraction of high-dimensional quantitative features from MR images, offering a more objective means of characterizing tissue heterogeneity and subtle pathological alterations. Several prior studies have demonstrated promising performance in differentiating NPC from benign nasopharyngeal hyperplasia [8–11]. However, most of these approaches treat lesions as homogeneous entities and do not explicitly model intralesional spatial heterogeneity, which may be particularly relevant in indeterminate Grade 3 lesions.

Habitat radiomics addresses this limitation by partitioning lesions into subregions based on voxel-level characteristics, thereby capturing spatial heterogeneity within a lesion [12–14]. Although habitat-based approaches have been explored in NPC for predicting treatment response and therapeutic outcomes [15–19], their application to resolving indeterminate Grade 3 nasopharyngeal lesions has not been established. Given the inherent spatial heterogeneity of early carcinoma, we hypothesized that MRI-based habitat radiomics may provide complementary information for differentiating diagnostically challenging Grade 3 nasopharyngeal lesions. Therefore, this study aimed to evaluate the feasibility of MRI-based habitat radiomics for distinguishing lymphoid hyperplasia from early-stage carcinoma and to explore its potential role in assisting radiologists in challenging diagnostic scenarios.

## Methods

### Patients

This retrospective, single-center diagnostic study was approved by the institutional review board (Approval No. LYREC2024-k165-01), and the requirement for written informed consent was waived in accordance with the Declaration of Helsinki. From March 2016 to January 2025, consecutive patients who underwent contrast-enhanced MRI of the nasopharynx followed by endoscopic biopsy were screened. Eligible patients had an MRI performed prior to biopsy and histopathological confirmation of either NPLH or early-stage NPC. Only lesions classified as Grade 3 according to the MRI grading system proposed by King et al. [3] were included. Within this grading framework, Grade 3 denotes asymmetric nasopharyngeal wall thickening and/or focal signal alteration without definite expansile growth or overt deep tissue invasion. Importantly, Grade 3 represents an imaging-based category of diagnostic uncertainty rather than a pathological diagnosis. Lesions graded as 2 (benign imaging features) or 4–5 (malignant imaging features) were excluded. Cases with incomplete, unavailable, or poor-quality MRI were also excluded. All MRI examinations were independently reviewed by two radiologists with 11 and 12 years of experience in head and neck imaging, respectively, who were blinded to histopathological results. Disagreements were resolved by consensus prior to model development. A total of 138 patients met the eligibility criteria and were randomly assigned to a training cohort (*n* = 97) and an independent test cohort (*n* = 41) using a 7:3 ratio. The patient selection process is illustrated in Fig. S1.

### Nasopharynx MRI protocols

All patients underwent a standardized nasopharyngeal MRI protocol, with minor adjustments based on scanner specifications. MRI scans were acquired on systems from multiple vendors, including Signa HDxt 1.5T (GE Healthcare), Prodiva 1.5T (Philips Healthcare), and Ingenia CX 3.0T (Philips Healthcare). Transverse sequences included T1-weighted imaging (T1WI), T2-weighted imaging (T2WI), and contrast-enhanced T1-weighted imaging (CE-T1WI). CE-T1WI images were obtained after intravenous administration of gadopentetate dimeglumine at a dose of 0.2 mmol/kg at 2 mL/s, followed by a 30 mL saline flush. Detailed sequence parameters are provided in Table S1. All images were exported in Digital Imaging and Communications in Medicine format and retrieved from the Picture Archiving and Communication System for subsequent analysis.

### Clinicoradiological features evaluation

Clinical data were retrieved from the Electronic Medical Record System, including age, sex, smoking history, alcohol consumption, hypertension, and presenting symptoms commonly associated with nasopharyngeal disease, such as blood-tinged nasal discharge, postnasal blood-streaked sputum, ear fullness, conductive hearing loss or tinnitus, and rhinobyon.

Two radiologists with 11 and 12 years of experience in head and neck imaging independently evaluated the MRI features. Both radiologists were blinded to the histopathological results, and discrepancies were resolved by consensus. Image interpretation was performed according to the MRI grading system proposed by King et al. [3], which stratifies nasopharyngeal findings into five categories based on the radiologic likelihood of carcinoma. Grade 1 corresponds to a normal nasopharyngeal appearance. Grade 2 denotes imaging findings considered probably benign, most commonly consistent with lymphoid hyperplasia. Grade 4 indicates imaging features suspicious for carcinoma, and Grade 5 reflects imaging findings considered probably carcinoma. This grading framework represents a radiologic likelihood assessment rather than a pathological diagnosis. Grade 3 is defined as an indeterminate imaging category characterized by asymmetric nasopharyngeal wall thickening and/or focal signal alteration without definitive invasive features, such as skull base erosion, parapharyngeal extension, or expansile mass formation. Importantly, Grade 3 does not exclude the presence of imaging features commonly associated with benign hyperplasia. In clinical practice, findings such as retention cysts, air bubbles, or vertically oriented mucosal stripes may coexist with asymmetric thickening or atypical signal changes. When benign-associated and suspicious imaging features overlap in the absence of unequivocal malignant characteristics, the lesion is categorized as Grade 3. Thus, Grade 3 represents a radiologic gray zone of diagnostic uncertainty rather than a distinct disease entity.

Asymmetry was not used as a standalone diagnostic criterion. Wall thickness asymmetry was assessed by visually comparing the left and right nasopharyngeal walls in the midsagittal plane and interpreted in conjunction with enhancement characteristics, internal architecture, and associated findings. Contrast enhancement on CE-T1WI was categorized as mild (less than normal mucosa), moderate (similar to normal mucosa), or marked (greater than normal mucosa). Enhancement patterns were classified as homogeneous or heterogeneous. Retention cysts were defined as lesions hypointense on T1WI and CE-T1WI and markedly hyperintense on T2WI. Air bubbles were identified as a highly hypointense signal on both T1WI and T2WI. Vertical stripes were defined as alternating linear hypointense and hyperintense bands within the unilateral mucosa on CE-T1WI. Associated findings, including middle ear effusion and cervical lymphadenopathy, were documented. Middle ear effusion was identified on T2WI. Lymph node involvement was assessed according to the criteria proposed by King et al. [3]. A node was considered metastatic if it met any of the following criteria: a minimum axial diameter of ≥ 6 mm for retropharyngeal nodes, ≥ 11 mm for jugulodigastric nodes, ≥ 10 mm for other cervical nodes, the presence of a cluster of three or more nodes with a minimal axial diameter of ≥ 8 mm, or imaging evidence of nodal necrosis or extracapsular spread. Representative MRI examples for different grading categories (Grades 2, 3, and 5) are shown in Fig. S2 to illustrate the imaging spectrum and the inherent ambiguity of indeterminate lesions.

### Lesion segmentation, habitat construction, and feature extraction

All MR images underwent standardized preprocessing, including N4 bias field correction to reduce intensity inhomogeneity, image-level Z-score normalization to standardize signal intensity, histogram discretization into 25 bins, and resampling to isotropic voxels of 1 × 1 × 1 mm³ using B-spline interpolation.

The entire nasopharyngeal lesion was manually segmented slice-by-slice on contrast-enhanced T1-weighted images using ITK-SNAP (version 3.6.0) by a radiologist with 11 years of experience in head and neck imaging, who was blinded to pathological results. To assess segmentation reproducibility, the same radiologist repeated the segmentation after a one-month interval in 30 randomly selected cases, and a second radiologist with 12 years of experience independently performed segmentation in the same subset. Intra- and interobserver reproducibility of extracted radiomics features was quantified using the intraclass correlation coefficient (ICC) based on a two-way random-effects model with absolute agreement. Features with ICC values < 0.80 were excluded from further analysis.

Radiomics features were extracted from the whole-lesion volume of interest using the open-source PyRadiomics package (version 3.0.1) [20], including first-order statistics, shape descriptors, and texture features. These features constituted the conventional radiomics feature set. For habitat radiomics analysis, voxel-level radiomic features within the segmented lesion were clustered using a Gaussian mixture model to identify spatially distinct subregions reflecting intralesional heterogeneity. The optimal number of clusters (range, 2–8) was determined using the Bayesian Information Criterion. Based on this data-driven selection, each lesion was partitioned into three intralesional subregions: subregion 1 (sub1), subregion 2 (sub2), and subregion 3 (sub3). Because geometric descriptors are not meaningful for internally clustered regions, shape features were excluded from subregion-level feature extraction. Radiomics features derived from these subregions were defined as habitat radiomics features.

**Fig. 1:**
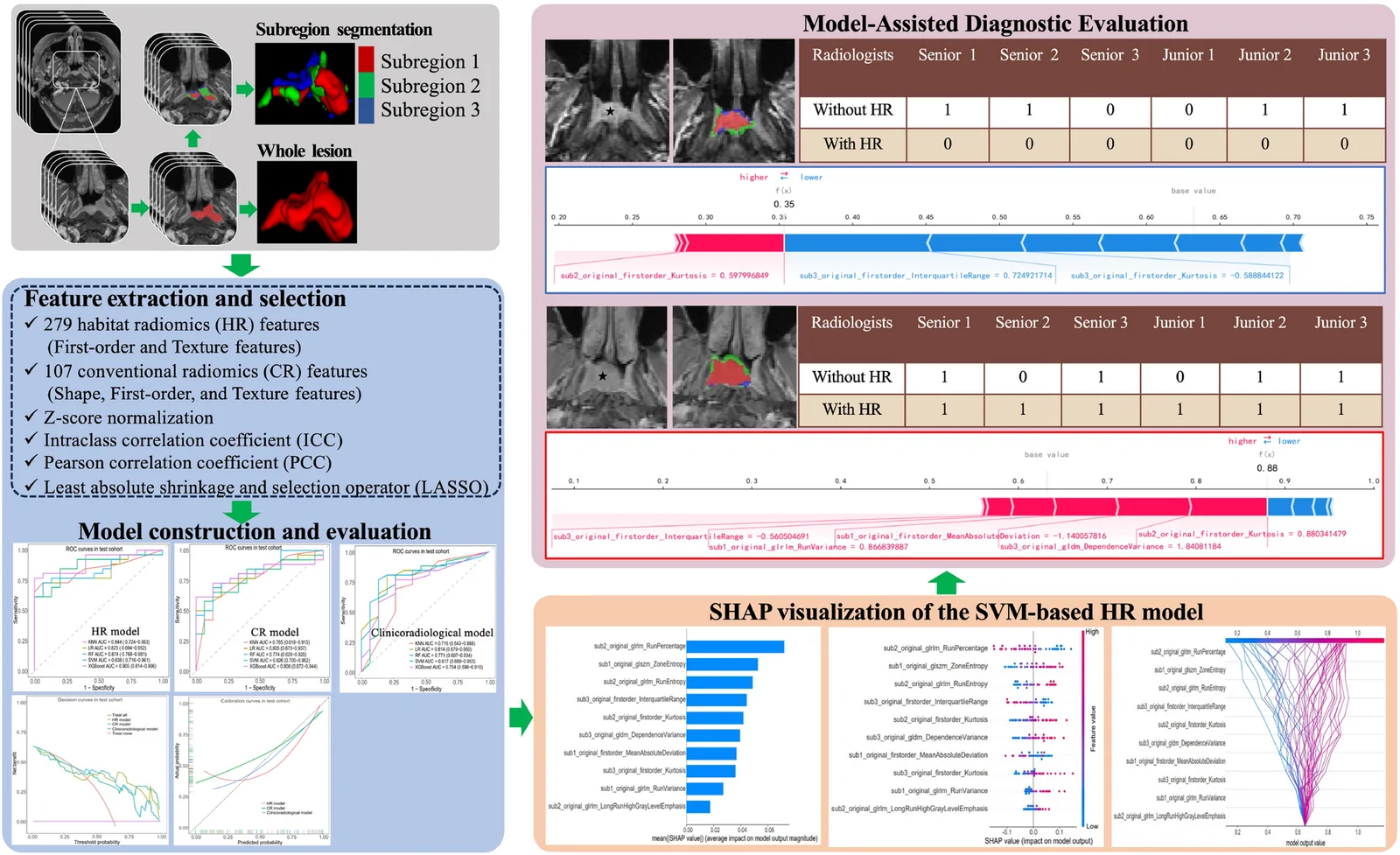
A brief workflow of this research

After feature extraction, conventional and habitat radiomics features were standardized using Z-score normalization based on the mean and standard deviation of the training cohort, and the same parameters were applied to the independent test cohort. To reduce redundancy and multicollinearity, pairwise Pearson correlation coefficients were calculated within the training cohort, and one feature from each pair with a correlation coefficient > 0.75 was removed. Subsequently, least absolute shrinkage and selection operator regression with 10-fold cross-validation was applied in the training cohort to identify the most informative features for model construction. The resulting feature-selection procedure was performed exclusively in the training cohort, and the selected feature set was then applied to the independent test cohort. A schematic overview of the workflow is presented in Fig. 1.

### Model development, validation, and interpretability

Three types of predictive models were constructed: a habitat radiomics model based on subregional features, a conventional radiomics model based on whole-lesion features, and a clinicoradiological model incorporating clinical and MRI-based imaging variables. All feature selection, clinicoradiological variable evaluation, and classifier selection procedures were performed exclusively within the training cohort. The independent test cohort was not used during model development or classifier selection and was reserved for final model evaluation. Five machine-learning classifiers—K-nearest neighbors, logistic regression, random forest, support vector machine, and extreme gradient boosting—were evaluated using 10-fold cross-validation. Classifier evaluation and parameter setting were confined to the training cohort. The final classifier was selected before test-cohort evaluation, and the independent test cohort was used only once for final performance assessment. Model performance was assessed using the area under the receiver operating characteristic curve (AUC), calibration curves, and decision curve analysis. Model interpretability was examined using SHapley Additive exPlanations (SHAP) to quantify feature contributions and to visualize both cohort-level and individual-level prediction behavior.

### Model-assisted diagnostic evaluation

Six radiologists (three seniors with 11–20 years of experience and three juniors with 2–8 years of experience in head and neck MRI) independently reviewed MR images of the test cohort in two rounds, presented in randomized order, to assess the model’s diagnostic assistance. In Round 1, radiologists evaluated clinical information and multi-sequence MRI findings and classified lesions according to established MRI diagnostic criteria, including the grading framework proposed by King et al. [3]. Asymmetric nasopharyngeal mucosal thickening was not used as a standalone diagnostic criterion but was interpreted in conjunction with enhancement patterns, internal architecture, and associated imaging findings. In Round 2, radiologists repeated the same assessments with access to the model’s dichotomous classification output, which labeled lesions as NPC-positive if the predicted probability exceeded the predefined cut-off. All radiologists were unaware of each other’s assessments, original diagnostic reports, and final pathological results. To reduce potential recall bias, case order was independently randomized for each assessment session, assessments were separated by a one-week interval, and no feedback regarding prior decisions or pathological results was provided between sessions. Model-assisted assessments were performed separately for the clinicoradiological, conventional radiomics, and habitat radiomics models.

### Statistical analysis

Categorical variables were compared using the Chi-square or Fisher’s exact test. The reproducibility of radiomics features was evaluated using the ICC. The SHAP method was used to assess the contributions of radiomics features. All statistical analyses were performed using IBM SPSS Statistics version 26.0 and R software version 4.4.2. All hypothesis tests were two-tailed, and a two-tailed *P* value < 0.05 was considered statistically significant.

## Results

### Clinical and MRI characteristics

The demographic, clinical, and MRI characteristics of patients in the training and test cohorts are summarized in Table 1. In the training cohort, the early-stage NPC group had a significantly higher proportion of male patients (*P* = 0.008) and cigarette smokers (*P* = 0.042) compared with the NPLH group. Regarding MRI features, nasopharyngeal mucosal thickness was significantly greater in early-stage NPC than in NPLH (*P* < 0.001). Vertical stripes along the nasopharyngeal mucosa were observed exclusively in the NPLH group and were absent in early-stage NPC. Retention cysts and intralesional bubbles were more frequently identified in NPLH (*P* = 0.003). Cervical lymph node enlargement was more common in early-stage NPC, whereas only four cases in the NPLH group showed this finding (*P* = 0.007).

**Table 1:** Clinical and MR characteristics of Grade 3 nasopharyngeal lesions with final histopathologic diagnosis of NPLH and early-stage NPC in the training and test cohorts

Characteristics	Training cohort (*n* = 97)			Test cohort (*n* = 41)			*P*_inter_^§^
	NPLH (*n* = 35)	Early-stage NPC (*n* = 62)	*P*_intra_^‡^	NPLH (*n* = 15)	Early-stage NPC (*n* = 26)	*P*_intra_^‡^	
Age			0.199			1.000	0.281
0, ≤ 49 years	23 (65.71%)	31 (50.00%)		7 (46.67%)	11 (42.31%)		
1, > 49 years	12 (34.29%)	31 (50.00%)		8 (53.33%)	15 (57.69%)		
Gender			0.008			0.667	0.543
0, Female	23 (65.71%)	22 (35.48%)		7 (46.67%)	9 (34.62%)		
1, Male	12 (34.29%)	40 (64.52%)		8 (53.33%)	17 (65.38%)		
Cigarette smoking			0.042			1.000	0.228
0, Absent	31 (88.57%)	42 (67.74%)		10 (66.67%)	16 (61.54%)		
1, Present	4 (11.43%)	20 (32.26%)		5 (33.33%)	10 (38.46%)		
Alcohol consumption			0.127			1.000	0.891
0, Absent	26 (74.29%)	35 (56.45%)		10 (66.67%)	17 (65.38%)		
1, Present	9 (25.71%)	27 (43.55%)		5 (33.33%)	9 (34.62%)		
Hypertension			0.318			1.000	0.799
0, Absent	33 (94.29%)	53 (85.48%)		13 (86.67%)	22 (84.62%)		
1, Present	2 (5.71%)	9 (14.52%)		2 (13.33%)	4 (15.38%)		
Blood tinged nasal discharge			0.481			0.72	0.037
0, Absent	33 (94.29%)	55 (88.71%)		12 (80.00%)	19 (73.08%)		
1, Present	2 (5.71%)	7 (11.29%)		3 (20.00%)	7 (26.92%)		
Postnasal blood streaked sputum			0.128			0.671	1.000
0, Absent	27 (77.14%)	37 (59.68%)		11 (73.33%)	16 (61.54%)		
1, Present	8 (22.86%)	25 (40.32%)		4 (26.67%)	10 (38.46%)		
Ear fullness, conductive hearing loss or tinnitus			0.321			0.543	0.755
0, Absent	33 (94.29%)	54 (87.10%)		13 (86.67%)	25 (96.15%)		
1, Present	2 (5.71%)	8 (12.90%)		2 (13.33%)	1 (3.85%)		
Rhinobyon			1.000			1.000	0.598
0, Absent	29 (82.86%)	51 (82.26%)		13 (86.67%)	23 (88.46%)		
1, Present	6 (17.14%)	11 (17.74%)		2 (13.33%)	3 (11.54%)		
Lesion volume			< 0.001			0.258	0.264
0,≤2648 mm^3^	25 (71.43%)	20 (32.26%)		11 (73.33%)	13 (50.00%)		
1, > 2648 mm^3^	10 (28.57%)	42 (67.74%)		4 (26.67%)	13 (50.00%)		
Lesion location			0.927			1.000	0.361
0, Posterior wall	13 (37.14%)	25 (40.32%)		4 (26.67%)	8 (30.77%)		
1, Unilateral posterolateral wall	22 (62.86%)	37 (59.68%)		11 (73.33%)	18 (69.23%)		
Enhancement degree			0.050			0.211	1.000
0, Slight	14 (40.00%)	39 (62.90%)		6 (40.00%)	17 (65.38%)		
1, Moderate	21 (60.00%)	23 (37.10%)		9 (60.00%)	9 (34.62%)		
Enhancement pattern			0.003			0.053	0.700
0, Homogeneous enhancement	19 (54.29%)	52 (83.87%)		9 (60.00%)	23 (88.46%)		
1, Presence of vertical stripes, nasopharyngeal bubble, retention cysts, or inhomogeneous enhancement	16 (45.71%)	10 (16.13%)		6 (40.00%)	3 (11.54%)		
Cervical lymph nodes enlargement			0.007			0.033	0.608
0, Absent	30 (85.71%)	35 (56.45%)		14 (93.33%)	16 (61.54%)		
1, Present	5 (14.29%)	27 (43.55%)		1 (6.67%)	10 (38.46%)		
Retropharyngeal lymph nodes enlargement			0.707			0.278	0.750
0, Absent	33 (94.29%)	56 (90.32%)		15 (100.00%)	22 (84.62%)		
1, Present	2 (5.71%)	6 (9.68%)		0 (0.00%)	4 (15.38%)		
Middle ear effusion			0.364			1.000	1.000
0, Absent	31 (88.57%)	49 (79.03%)		13 (86.67%)	21 (80.77%)		
1, Present	4 (11.43%)	13 (20.97%)		2 (13.33%)	5 (19.23%)		

*MR* magnetic resonance, *NPLH* nasopharyngeal lymphoid hyperplasia, *NPC* nasopharyngeal carcinoma

*P*_intra_^‡^: *p*-value between the NPLH and early-stage NPC groups. *P*_inter_^§^: *p*-value between the training and test cohorts

Univariate logistic regression analysis identified gender (*P* = 0.005), cigarette smoking (*P* = 0.029), lesion volume (*P* < 0.001), enhancement degree (*P* = 0.031), enhancement pattern (*P* = 0.002), and cervical lymph node enlargement (*P* = 0.005) as significantly associated with early-stage NPC (Table S2). Although gender, cigarette smoking, and enhancement degree were not statistically significant in the multivariate logistic regression analysis, they were included in the model based on clinical importance and prior clinical experience.

### Habitat subregion distribution

In Fig. 1, the subregions are arranged in concentric layers: red indicates sub1, green indicates sub2, and blue indicates sub3. Sub1 was predominantly located in the central portion of the lesion and demonstrated relatively higher signal intensity. Sub2 showed intermediate signal intensity and was distributed across both superficial and deeper components. Sub3 was primarily located at the lesion periphery adjacent to normal nasopharyngeal mucosa and demonstrated relatively lower signal intensity.

### Feature extraction and selection

Habitat subregions were generated using Gaussian mixture model-based clustering. Because these internally clustered subregions did not correspond to the original lesion morphology, shape-based features were not extracted from individual subregions. From contrast-enhanced T1-weighted images, 279 habitat radiomics features and 107 conventional radiomics features were initially extracted. After feature selection, 10 habitat radiomics features and 13 conventional radiomics features were retained for model construction (Fig. S3).

### Model construction and evaluation

**Fig. 2:**
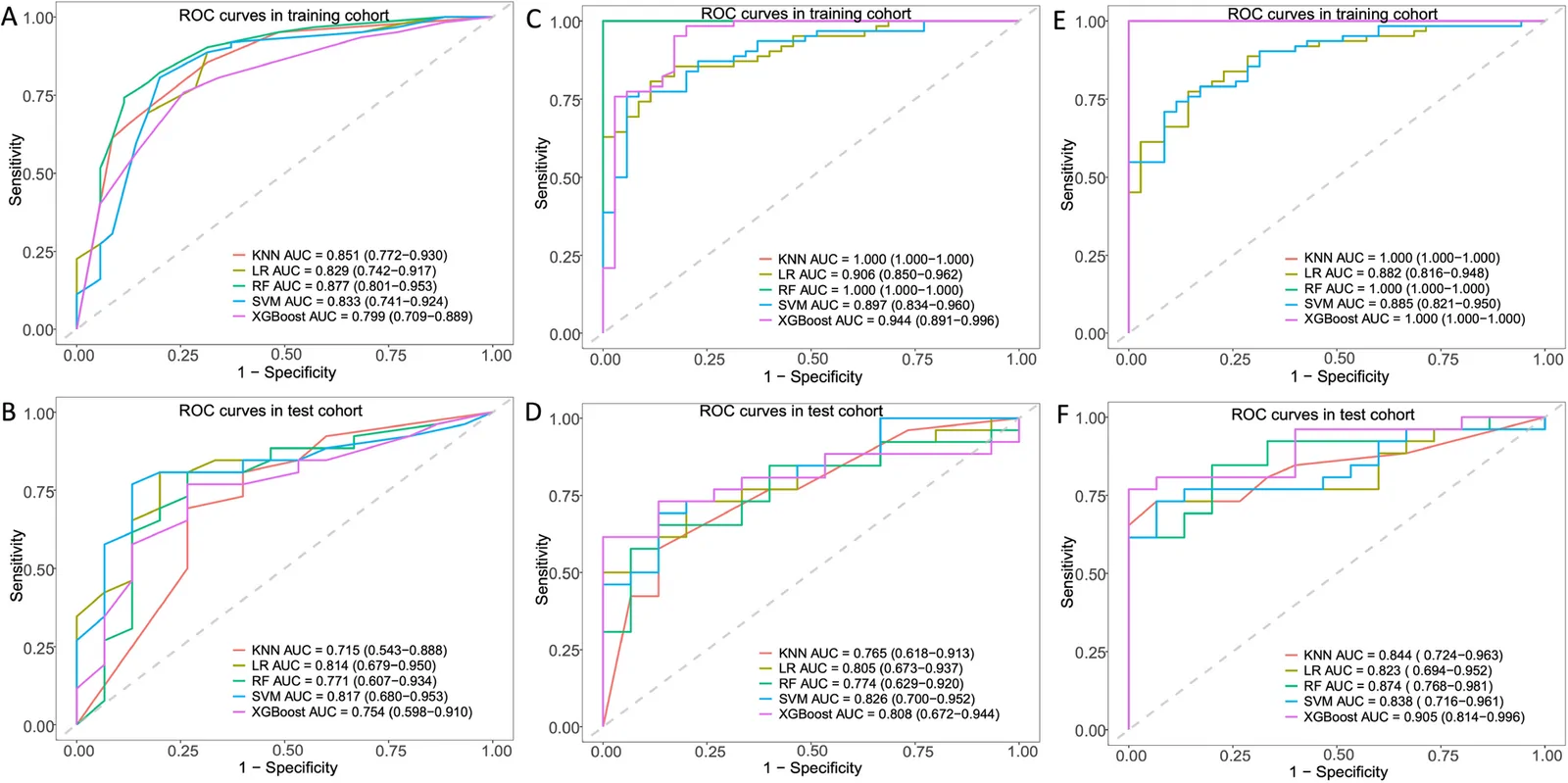
AUC of ROC curves for differentiating indeterminate (Grade 3) nasopharyngeal lesions (NPLH vs. early-stage NPC) using five machine learning classifiers. (**A**,** B**) Clinicoradiological model; (**C**,** D**) Conventional radiomics model; (**E**,** F**) Habitat radiomics model. AUC, area under the curve; KNN, K-nearest neighbors; LR, logistic regression; MRI, magnetic resonance imaging; NPC, nasopharyngeal carcinoma; NPLH, nasopharyngeal lymphoid hyperplasia; RF, random forest; ROC, receiver operating characteristic curve; SVM, support vector machine; XGBoost, extreme gradient boosting

Classifier selection was performed exclusively within the training cohort using 10-fold cross-validation. Among the evaluated algorithms, the support vector machine classifier demonstrated stable cross-validated performance and was selected for final model construction. In the independent test cohort, the habitat radiomics model based on the support vector machine classifier achieved an AUC of 0.838 (95% confidence interval [CI]: 0.716–0.961), which was numerically but not significantly higher than those of the conventional radiomics model (AUC = 0.826, 95% CI: 0.700-0.952) and the clinicoradiological model (AUC = 0.817, 95% CI: 0.680–0.953) (Fig. 2). Differences in AUC among the three models were not statistically significant (all *P* > 0.05). All models demonstrated adequate calibration and yielded positive clinical net benefit (Fig. S4).

### Model-assisted diagnostic evaluation

The diagnostic performance of radiologists with and without model assistance is presented in Table 2 and Table S3. Without model assistance, AUC values ranged from 0.632 to 0.828. Junior radiologists demonstrated lower AUCs (0.632–0.704) compared with senior radiologists (0.665–0.828). Accuracy ranged from 0.659 to 0.854, and sensitivity (0.731–0.923) and specificity (0.533–0.733) varied among radiologists. With the assistance of the clinicoradiological model and conventional radiomics model, most radiologists showed modest improvements in AUC. DeLong tests indicated no significant differences in performance between radiologists with and without model assistance (all *P* > 0.05). With the assistance of the habitat radiomics model, radiologists showed descriptive increases in AUC (0.828–0.928), accuracy (0.854–0.927), sensitivity (0.846–0.962), and specificity (0.733–0.933). DeLong tests indicated that statistically significant improvements relative to pre-assistance were observed mainly among junior radiologists (*P* < 0.05).

**Table 2:** Diagnostic performance of radiologists with and without model assistance in the test cohort

		AUC (95%CI)	Accuracy	Sensitivity	Specificity	PPV	NPV
Without model	Senior 1	0.723 (0.577–0.870)	0.756	0.846	0.600	0.786	0.692
	Senior 2	0.665 (0.510–0.820)	0.683	0.731	0.600	0.760	0.562
	Senior 3	0.828 (0.701–0.955)	0.854	0.923	0.733	0.857	0.846
	Junior 1	0.704 (0.554–0.854)	0.732	0.808	0.600	0.778	0.643
	Junior 2	0.632 (0.475–0.789)	0.659	0.731	0.533	0.731	0.533
	Junior 3	0.699 (0.548–0.850)	0.707	0.731	0.667	0.792	0.588
With clinicoradiological model	Senior 1	0.746 (0.608–0.885)	0.732	0.692	0.800	0.857	0.600
	Senior 2	0.699 (0.548–0.850)	0.707	0.731	0.667	0.792	0.588
	Senior 3	0.823 (0.697–0.949)	0.829	0.846	0.800	0.88	0.750
	Junior 1	0.727 (0.587–0.867)	0.707	0.654	0.800	0.85	0.571
	Junior 2	0.671 (0.519–0.822)	0.707	0.808	0.533	0.75	0.615
	Junior 3	0.732 (0.587–0.877)	0.732	0.731	0.733	0.826	0.611
With CR model	Senior 1	0.823 (0.697–0.949)	0.829	0.846	0.800	0.880	0.750
	Senior 2	0.751 (0.609–0.894)	0.756	0.769	0.733	0.833	0.647
	Senior 3	0.818 (0.697–0.939)	0.805	0.769	0.867	0.909	0.684
	Junior 1	0.779 (0.653–0.906)	0.756	0.692	0.867	0.900	0.619
	Junior 2	0.790 (0.654–0.925)	0.805	0.846	0.733	0.846	0.733
	Junior 3	0.746 (0.608–0.885)	0.732	0.692	0.800	0.857	0.600
With HR model	Senior 1	0.842 (0.720–0.964)	0.854	0.885	0.800	0.885	0.800
	Senior 2	0.828 (0.701–0.955)	0.854	0.923	0.733	0.857	0.846
	Senior 3	0.928 (0.845-1.000)	0.927	0.923	0.933	0.960	0.875
	Junior 1	0.895 (0.792–0.998)	0.902	0.923	0.867	0.923	0.867
	Junior 2	0.847 (0.726–0.969)	0.878	0.962	0.733	0.862	0.917
	Junior 3	0.856 (0.743–0.970)	0.854	0.846	0.867	0.917	0.765

*AUC* area under the curve, *CI* confidence interval, *CR* conventional radiomics, *HR* habitat radiomics, *NPV* negative predictive value, *PPV* positive predictive value, Without model, physician-independent diagnosis; With CR model, CR modeling aids in the diagnosis; With HR model, HR modeling aids in the diagnosis

### SHAP visual analysis

**Fig. 3:**
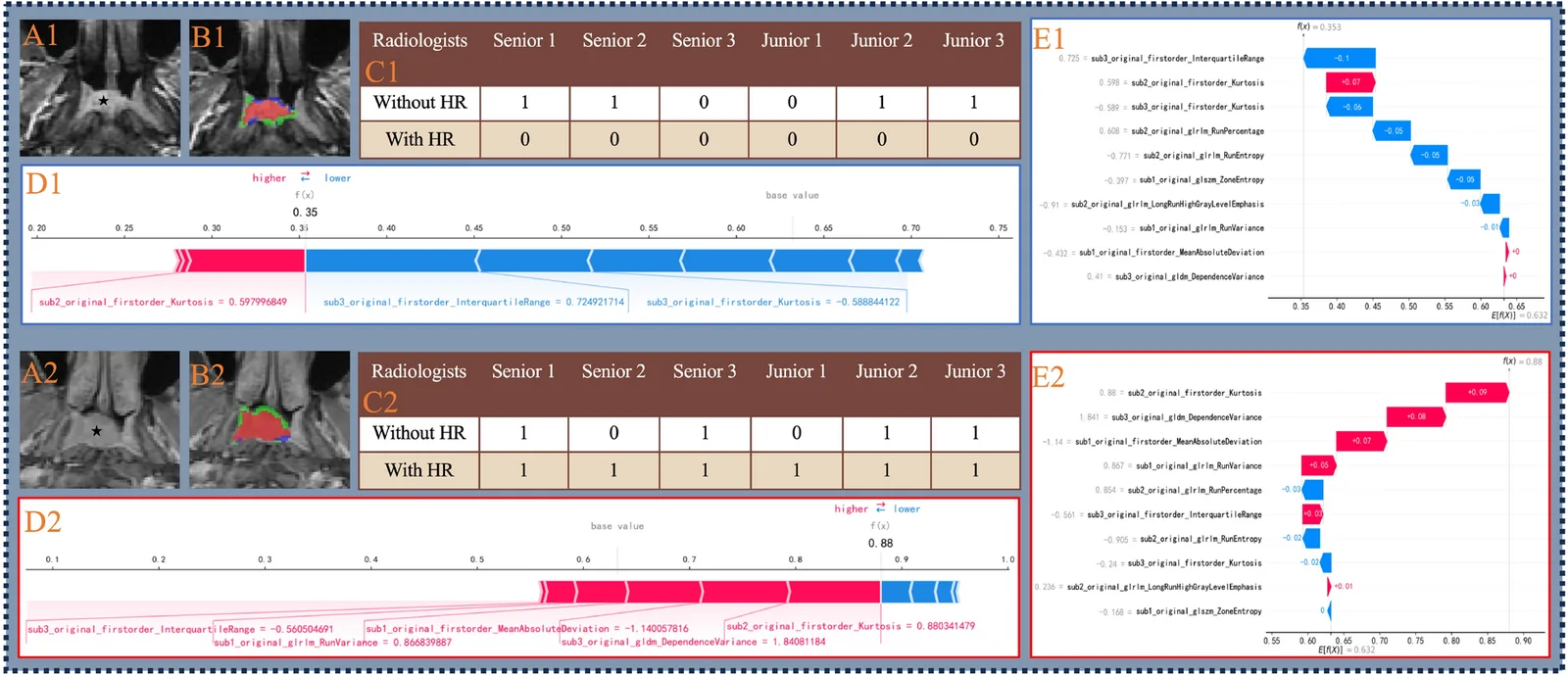
Representative cases of Grade 3 nasopharyngeal lesions with corresponding SHAP visualizations from the habitat radiomics model and radiologist interpretations with and without model assistance. Patient 1: (**A**1, **B**1) Axial CE-T1WI demonstrates a nasopharyngeal lesion categorized as Grade 3 (indeterminate imaging appearance according to the CUHK MRI grading system). At independent reading, 66.7% of radiologists favored early-stage NPC (**C**1). Histopathology confirmed nasopharyngeal lymphoid hyperplasia (NPLH). The habitat radiomics model assigned a higher probability to NPLH (**D**1, **E**1), illustrated by the SHAP force and waterfall plots. When the model assistance was provided, fewer radiologists favored NPC. Patient 2: (**A**2, **B**2) Axial CE-T1WI demonstrates a lesion categorized as Grade 3 (indeterminate imaging appearance). At independent reading, 33.3% of radiologists favored NPLH (**C**2). Histopathology confirmed early-stage NPC. The habitat radiomics model assigned a higher probability to NPC (**D**2, **E**2). After reviewing the model output, more radiologists favored NPC. CE-T1WI, contrast-enhanced T1-weighted imaging; CUHK, Chinese University of Hong Kong; NPC, nasopharyngeal carcinoma; NPLH, nasopharyngeal lymphoid hyperplasia; SHAP, SHapley Additive exPlanations

We calculated overall and individual SHAP values for the habitat radiomics model to enhance interpretability. The SHAP bar chart (Fig. S5A) presents the ten most important features, along with their corresponding weights and subregional distributions. The SHAP summary plot (Fig. S5B) shows the impact of these ten features on the prediction outcomes across all participants, reflecting their overall contribution to early-stage NPC classification. The SHAP decision plot (Fig. S5C) visualizes the cumulative effect of each feature on the model’s decision-making process for all participants. In individual case visualizations, Figs. 3A-C illustrate two cases in which the habitat radiomics model correctly classified NPLH and early-stage NPC, and show the diagnostic changes made by six radiologists before and after applying the habitat radiomics model. Furthermore, the SHAP force plot (Fig. 3D) provides a detailed breakdown of the key feature categories that influence the model’s prediction for individual lesions, indicating the direction and magnitude of each feature’s impact on the final classification. Additionally, the SHAP waterfall plot (Fig. 3E) illustrates how each feature contributes, step by step, to the final prediction for a single participant, providing a clear visualization of the model’s decision path.

## Discussion

This study evaluated the feasibility of a habitat radiomics model for differentiating indeterminate nasopharyngeal lesions (Grade 3) into NPLH or early-stage NPC. Within the Chinese University of Hong Kong MRI grading system, Grade 3 represents an imaging-defined state of diagnostic uncertainty—it does not correspond to a specific pathological diagnosis but is a gray zone where benign processes (such as NPLH) and early malignancies (such as early-stage NPC) overlap. The habitat radiomics model showed feasible performance in distinguishing between these two conditions, although its standalone performance was not statistically superior to that of the comparator models. Model-assisted reading was associated with improved diagnostic performance mainly among junior radiologists.

Previous studies using radiomics or deep learning approaches typically treat lesions as homogeneous entities [8–10]. In contrast, the habitat radiomics approach incorporated intralesional spatial heterogeneity by partitioning lesions into subregions. This method is particularly relevant for Grade 3 lesions, where conventional MRI features may not clearly distinguish between NPLH and early-stage NPC. In the independent test cohort, the habitat radiomics model showed numerically higher AUC values than the conventional radiomics and clinicoradiological models, but the differences were not statistically significant. These findings do not demonstrate definitive model superiority. Instead, spatial heterogeneity may provide complementary information and support less-experienced readers in indeterminate cases.

Clinicoradiological analysis demonstrated measurable differences between early-stage NPC and NPLH within the Grade 3 cohort. Male sex and smoking history were more frequent in early-stage NPC, consistent with prior reports indicating a higher incidence in men [1, 21] and an association between heavy smoking and NPC risk, particularly in genetically susceptible individuals [22, 23]. Lesion volume and cervical lymph node enlargement were also more frequently observed in early-stage NPC, while imaging features typically associated with benign hyperplasia—such as vertical mucosal stripes, retention cysts, and intralesional air bubbles—were more common in NPLH [1, 7, 24]. These findings indicate that, even within an imaging-defined indeterminate category, certain clinical and structural characteristics remain differentially distributed. However, the substantial overlap in features underscores the intrinsic diagnostic ambiguity of Grade 3 lesions and the limitations of relying solely on conventional MRI.

SHAP analysis showed that radiomics features derived from all three intralesional subregions contributed to model predictions, rather than demonstrating dominance of a specific subregion. Among the top-ranked features, first-order features such as sub2_original_firstorder_Kurtosis and sub3_original_firstorder_InterquartileRange describe the distribution shape and dispersion of voxel intensities, whereas texture features such as sub3_original_gldm_DependenceVariance and sub1_original_glrlm_RunVariance reflect local signal dependence and run-length variability. These features may capture differences in intensity distribution, enhancement heterogeneity, and internal architectural complexity across different lesion compartments. From a spatial perspective, sub1 approximates the lesion core, whereas sub2 and sub3 represent progressively more superficial compartments [25]. Signal and textural variation along internal interfaces and lesion margins may therefore provide information beyond whole-lesion analysis [26, 27]. This interpretation is broadly consistent with known MRI differences between NPLH and early-stage NPC, particularly in lesion architecture and enhancement heterogeneity. However, these radiomics features should be interpreted as quantitative imaging descriptors rather than direct histopathological correlates.

Radiologist-assistance analysis further contextualized model performance. Without assistance, junior radiologists demonstrated lower diagnostic performance and greater variability than senior radiologists. Assistance from the habitat radiomics model was associated with descriptive improvements in AUC, sensitivity, and overall accuracy, with greater gains among junior radiologists. In contrast, the clinicoradiological and conventional radiomics models yielded smaller and non-significant changes. These findings suggest that habitat-based quantitative assessment may provide complementary support for less-experienced readers in challenging gray-zone cases, although clinical implementation would require prospective validation.

Misclassified cases were primarily observed in lesions exhibiting overlapping imaging characteristics between early-stage NPC and NPLH. Subtle early-stage NPC lesions with moderate enhancement and limited volumetric expansion were occasionally classified as NPLH, whereas inflammatory or hypervascular NPLH lesions with pronounced asymmetry were sometimes classified as NPC. These patterns reflect the inherent biological and imaging overlap within Grade 3 lesions rather than systematic model bias, emphasizing the fundamental difficulty of distinguishing early malignant transformation from benign hyperplastic change using conventional MRI criteria alone.

This study has several limitations. First, this was a retrospective single-center study, and external prospective validation in multi-center cohorts is required before clinical translation or broader clinical application. MRI examinations were acquired using scanners from different vendors and field strengths over a long period, with a predominance of GE-acquired examinations and relatively few Philips/3.0T cases. Because ComBat or equivalent harmonization was not performed, scanner-related variability, temporal protocol drift, and vendor imbalance may affect the robustness and generalizability of the radiomics features. Second, the small test cohort limited statistical power, and the numerically higher AUC of the habitat radiomics model was not statistically significant compared with comparator models. Although feature selection, clinicoradiological variable evaluation, and classifier selection were confined to the training cohort, clinically guided variable retention, the Pearson redundancy threshold, and classifier selection may still introduce analytical flexibility. Therefore, future studies with pre-specified analysis plans are needed. Third, the reader-assistance findings were mainly observed among junior radiologists and may be influenced by presentation order, anchoring, recall, or differential trust in model outputs; these findings should therefore be considered exploratory and confirmed in prospective, fully randomized reader studies. Finally, segmentation was performed manually, which may introduce observer dependency despite satisfactory reproducibility. Automated or semi-automated segmentation in larger datasets may further improve robustness and reproducibility.

## Conclusion

In conclusion, Grade 3 nasopharyngeal lesions represent an imaging-defined state of diagnostic uncertainty, where radiological findings overlap between benign hyperplasia and early-stage carcinoma. Habitat radiomics analysis showed feasible performance for differentiating NPLH from early-stage NPC within this gray zone, although its standalone performance was not statistically superior to comparator models. It may provide complementary support mainly for junior radiologists in challenging diagnostic cases. 

## Supplementary Information

Supplementary Material 1.

## Data Availability

The datasets used and/or analyzed during the current study are available from the corresponding author upon reasonable request.

## Abbreviations

AUC: Area under the curve
CE-T1WI: Contrast-enhanced T1-weighted imaging
CI: Confidence interval
ICC: Intraclass correlation coefficient
MRI: Magnetic resonance imaging
NPC: Nasopharyngeal carcinoma
NPLH: Nasopharyngeal lymphoid hyperplasia
SHAP: SHapley Additive exPlanations
T1WI: T1-weighted imaging
T2WI: T2-weighted imaging
