# Supplementary material for: MR habitat radiomics for differentiating indeterminate (Grade 3) nasopharyngeal lesions: lymphoid hyperplasia versus early-stage nasopharyngeal carcinoma
Source: BMC Cancer. 2026 Jul 15;26:1208. doi: 10.1186/s12885-026-16524-y (PMC13647505; doi:10.1186/s12885-026-16524-y)
Supplement: Supplementary file 1 [file 12885_2026_16524_MOESM1_ESM.docx]

**Legend of supplementary tables**

| **Table S1** Magnetic resonance imaging protocol | | | | | | | | | | |
| --- | --- | --- | --- | --- | --- | --- | --- | --- | --- | --- |
| MR device | Number of patients | Tesla | Sequences (transverse) | Parameters | | | | | | |
|  |  |  |  | TR (ms) | TE (ms) | Matrix | FOV (mm) | Slices | Thickness(mm) | Scan time |
| GE Signa HDxt | 112 | 1.5 | T_1_WI | 380 | 14 | 320 × 224 | 240 × 240 | 18 | 3 | 2:55 |
|  |  |  | T_2_WI | 5000 | 55 | 288 × 192 | 240 × 240 | 18 | 3 | 2:45 |
|  |  |  | CE-T_1_WI | 540 | 11 | 320 × 192 | 240 × 240 | 18 | 3 | 3:32 |
| Philips Prodiva | 16 | 1.5 | T_1_WI | 585 | 15 | 276 × 197 | 220 × 220 | 24 | 3 | 2:33 |
|  |  |  | T_2_WI | 2756 | 84 | 292 × 212 | 220 × 220 | 24 | 3 | 2:45 |
|  |  |  | CE-T_1_WI | 585 | 15 | 276 × 191 | 220 × 220 | 24 | 3 | 2:15 |
| Philips Ingenia CX | 10 | 3.0 | T_1_WI | 632 | 8.7 | 308 × 255 | 200 × 200 | 24 | 3 | 1:37 |
|  |  |  | T_2_WI | 2500 | 100 | 332 × 248 | 200 × 200 | 24 | 3 | 2:15 |
|  |  |  | CE-T_1_WI | 503 | 8.1 | 400 × 278 | 220 × 200 | 24 | 3 | 2:16 |

CE-T1WI, contrast-enhanced T1-weighted images; FOV, field of view; T1WI, T1-weighted imaging; T2WI, T2-weighted imaging; TE, echo time; TR, repetition time.

| **Table S2** Univariate and multivariate analyses of clinical and MRI differences between Grade 3 nasopharyngeal lesions with final diagnoses of NPLH and early-stage NPC | | | | |
| --- | --- | --- | --- | --- |
| **Characteristics** | **Univariate** | | **Multivariate** | |
|  | **OR (95% CI)** | ***P* value** | **OR (95% CI)** | ***P* value** |
| **Age** |  | 0.137 |  |  |
| 0, ≤49 years | 1.000 |  |  |  |
| 1, >49 years | 1.917 (0.823-4.618) |  |  |  |
| **Gender** |  | 0.005 |  | 0.298 |
| 0, Female | 1.000 |  | 1.000 |  |
| 1, Male | 3.485 (1.484-8.540) |  | 1.928 (0.562-6.846) |  |
| **Cigarette smoking** |  | 0.029 |  | 0.596 |
| 0, Absent | 1.000 |  | 1.000 |  |
| 1, Present | 3.690 (1.246-13.65) |  | 1.545 (0.316-8.427) |  |
| **Alcohol consumption** |  | 0.084 |  |  |
| 0, Absent | 1.000 |  |  |  |
| 1, Present | 2.229 (0.920-5.751) |  |  |  |
| **Hypertension** |  | 0.205 |  |  |
| 0, Absent | 1.000 |  |  |  |
| 1, Present | 2.802 (0.670-19.13) |  |  |  |
| **Blood tinged nasal discharge** |  | 0.372 |  |  |
| 0, Absent | 1.000 |  |  |  |
| 1, Present | 2.100 (0.474-14.66) |  |  |  |
| **Postnasal blood streaked sputum** |  | 0.085 |  |  |
| 0, Absent | 1.000 |  |  |  |
| 1, Present | 2.280 (0.919-6.110) |  |  |  |
| **Ear fullness, conductive hearing loss or tinnitus** |  | 0.276 |  |  |
| 0, Absent | 1.000 |  |  |  |
| 1, Present | 2.444 (0.570-16.85) |  |  |  |
| **Rhinobyon** |  | 0.941 |  |  |
| 0, Absent | 1.000 |  |  |  |
| 1, Present | 1.042(0.357-3.297) |  |  |  |
| **Lesion volume** |  | <0.001 |  | 0.004 |
| 0, ≤2648 mm^3^ | 1.000 |  | 1.000 |  |
| 1, >2648 mm^3^ | 5.250 (2.177-13.48) |  | 4.906 (1.695-15.54) |  |
| **Lesion location** |  | 0.758 |  |  |
| 0, Posterior wall | 1.000 |  |  |  |
| 1, Unilateral posterolateral wall | 0.875 (0.367-2.041) |  |  |  |
| **Enhancement degree** |  | 0.031 |  | 0.150 |
| 0, Slight | 1.000 |  | 1.000 |  |
| 1, Moderate | 0.393 (0.165-0.911) |  | 0.465 (0.160-1.311) |  |
| **Enhancement pattern** |  | 0.002 |  | 0.010 |
| 0, Homogeneous enhancement | 1.000 |  | 1.000 |  |
| 1, Presence of vertical stripes, nasopharyngeal bubble, retention cysts, or inhomogeneous enhancement | 0.228 (0.086-0.580) |  | 0.211 (0.060-0.661) |  |
| **Cervical lymph nodes enlargement** |  | 0.005 |  | 0.012 |
| 0, Absent | 1.000 |  | 1.000 |  |
| 1, Present | 4.629 (1.693-14.99) |  | 5.133 (1.534-20.61) |  |
| **Retropharyngeal lymph nodes enlargement** |  | 0.500 |  |  |
| 0, Absent | 1.000 |  |  |  |
| 1, Present | 1.768(0.382-12.54) |  |  |  |
| **Middle ear effusion** |  | 0.242 |  |  |
| 0, Absent | 1.000 |  |  |  |
| 1, Present | 2.056(0.659-7.814) |  |  |  |

CI, conﬁdence interval; NPC, nasopharyngeal carcinoma; NPLH, nasopharyngeal lymphoid hyperplasia; OR, odds ratio.

| **Table S3** Comparison of radiologists' diagnostic performance with and without model assistance in the test cohort | | | | | |  |
| --- | --- | --- | --- | --- | --- | --- |
| **Radiologist** | **Model** |  | **Delong test (*P* value)** | | | |
|  |  | **Radiologist vs. Model** | | **Radiologist with model vs. Model** | **Without vs. With model** |  |
| Senior 1 | Clinicoradiological | 0.298 | | 0.474 | 0.787 |  |
|  | CR | 0.647 | | 0.284 | 0.132 |  |
|  | HR | 0.296 | | 0.317 | 0.203 |  |
| Senior 2 | Clinicoradiological | 0.080 | | 0.164 | 0.735 |  |
|  | CR | 0.290 | | 0.885 | 0.414 |  |
|  | HR | 0.118 | | 0.959 | 0.075 |  |
| Senior 3 | Clinicoradiological | 0.703 | | 0.717 | 0.937 |  |
|  | CR | 0.443 | | 0.071 | 0.899 |  |
|  | HR | 0.960 | | 0.172 | 0.097 |  |
| Junior 1 | Clinicoradiological | 0.334 | | 0.158 | 0.814 |  |
|  | CR | 0.503 | | 0.569 | 0.360 |  |
|  | HR | 0.257 | | 0.158 | 0.029 |  |
| Junior 2 | Clinicoradiological | 0.086 | | 0.082 | 0.713 |  |
|  | CR | 0.145 | | 0.624 | 0.032 |  |
|  | HR | 0.076 | | 0.598 | 0.033 |  |
| Junior 3 | Clinicoradiological | 0.294 | | 0.267 | 0.681 |  |
|  | CR | 0.422 | | 0.766 | 0.552 |  |
|  | HR | 0.180 | | 0.442 | 0.046 |  |

AUC, area under the curve; CI, confidence interval; CR, conventional radiomics; HR, habitat radiomics; Without model, physician-independent diagnosis; With CR model, CR modeling aids in the diagnosis; With HR, HR modeling aids in the diagnosis.

**Legend of supplementary figures**

**Fig. S1** Flowchart of the study population

**Fig. S2** Representative MR images interpreted according to the Chinese University of Hong Kong MR imaging grading system. **(A-C)** Axial T1-weighted **(A)**, T2-weighted **(B)**, and contrast-enhanced T1-weighted **(C)** images show symmetric nasopharyngeal mucosal thickening with a retention cyst (arrows) and vertically aligned dark and bright stripes (arrowheads), consistent with benign hyperplasia (Grade 2, probably benign hyperplasia). **(D-F)** Axial T1-weighted **(D)**, T2-weighted **(E)**, and contrast-enhanced T1-weighted **(F)** images show asymmetric nasopharyngeal mucosal thickening with a retention cyst (arrows), classified as indeterminate (Grade 3, indeterminate) and histopathologically confirmed nasopharyngeal lymphoid hyperplasia. Grade 3 represents an imaging-defined gray zone, not a specific diagnosis, where benign and malignant features overlap. **(G-I)** Axial T1-weighted **(G)**, T2-weighted **(H)**, and contrast-enhanced T1-weighted **(I)** images show nasopharyngeal carcinoma (Grade 5, probably nasopharyngeal carcinoma), characterized by asymmetric nasopharyngeal mucosal thickening with an adjacent metastatic retropharyngeal lymph node (arrows).

**Fig. S3** Feature selection using the least absolute shrinkage and selection operator (LASSO) algorithm. Lambda was determined via 10-fold cross-validation, and the selected features with non-zero coefficients are shown.

**Fig. S4** Calibration and clinical utility of SVM-based models.

**(A, B)** Calibration curves; **(C, D)** Decision curve analyses.

CR, conventional radiomics; HR, habitat radiomics; SVM, support vector machine.

**Fig. S5** SHAP analysis illustrates the contributions of features to early-stage NPC prediction. **(A)** SHAP bar chart: Features are ranked by their mean absolute SHAP values. **(B)** SHAP summary plot: Features are ranked by their mean SHAP values, highlighting their overall importance. **(C)** SHAP decision plot (all participants): SHAP values for all participants are summarized, and the overall feature contributions are demonstrated.

NPC, nasopharyngeal carcinoma; SHAP, SHapley Additive exPlanations.
